# Microinjection of Xenopus Laevis Oocytes

**DOI:** 10.3791/1106

**Published:** 2009-02-23

**Authors:** Sarah Cohen, Shelly Au, Nelly Panté

**Affiliations:** Department of Zoology, University of British Columbia - UBC

## Abstract

Microinjection of *Xenopus laevis* oocytes followed by thin-sectioning electron microscopy (EM) is an excellent system for studying nucleocytoplasmic transport. Because of its large nucleus and high density of nuclear pore complexes (NPCs), nuclear transport can be easily visualized in the *Xenopus* oocyte. Much insight into the mechanisms of nuclear import and export has been gained through use of this system (reviewed by Panté, 2006). In addition, we have used microinjection of *Xenopus* oocytes to dissect the nuclear import pathways of several viruses that replicate in the host nucleus.

Here we demonstrate the cytoplasmic microinjection of *Xenopus* oocytes with a nuclear import substrate. We also show preparation of the injected oocytes for visualization by thin-sectioning EM, including dissection, dehydration, and embedding of the oocytes into an epoxy embedding resin. Finally, we provide representative results for oocytes that have been microinjected with the capsid of the baculovirus *Autographa californica nucleopolyhedrovirus* (AcMNPV) or the parvovirus Minute Virus of Mice (MVM), and discuss potential applications of the technique.

**Figure:**
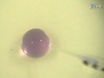


## Protocol

### Part 1: Preparation of *Xenopus* oocytes for microinjection

1. Place a small piece (about 2 cm) of *Xenopus laevis* ovary into a 50-ml conical tube containing 20 ml of collagenase solution (5 mg/ml collagenase in calcium-free modified Barth’s saline: 88 mM NaCl, 1 mM KCl, 0.82 mM MgSO_4_, 10 mM Hepes, pH 7.5). Collagenase is used to remove the follicle cells which surround the oocytes.
2. Place the tube on a shaker platform and rock it gently (at 100 RPM) for one hour. This time varies with different lots of collagenase. Thus after one hour, take a small sample of oocytes and examine them under a dissecting microscope. Properly de-folliculated oocytes should be well separated from one another, and it should be easy to insert a glass needle into the oocyte. If the oocytes are not sufficiently de-folliculated, they are left in the collagenase solution and checked again every five minutes.
3. Wash the oocytes three times with modified Barth’s saline (MBS: 88 mM NaCl, 1 mM KCl, 0.82 mM MgSO_4_, 0.33 mM Ca(NO_3_)_2_, 0.41 mM CaCl_2_, 10 mM Hepes, pH 7.5), and transfer them to a 100-mm diameter Petri dish containing MBS plus 1% penicillin/streptomycin. The oocytes are now ready to be microinjected. If microinjection will be performed on another day, store the oocytes at 4 °C for up to one week.
4. Using a dissecting microscope, select mature stage VI oocytes for microinjection. These oocytes are large, with good contrast between the black animal hemisphere and the creamy-colored vegetal hemisphere.
5. Transfer the mature stage VI oocytes into a microwell plate (Nunc, 10 µl well volume). This should be done carefully, using a 200-µl pipettor with a pipette tip that has been cut at the end to allow undisrupted suction of the oocytes.

### Part 2: Microinjection of *Xenopus* oocytes

1. Prepare the import substrate to be microinjected by adding a small amount of 1% bromphenol blue, to aid in the visualization of microinjection. For example, we add 1 µl of bromphenol blue to 10 µl of substrate.
2. Place a small strip of Parafilm on a 100-mm diameter Petri dish, and dispense a 5-µl drop of the solution to be injected on the Parafilm strip.
3. Fill a previously calibrated injection needle (obtained by pulling a 6.6-µl micropipette Drummond with the Inject+Matic Puller) with the solution to be injected. For this, the microinjector is set on aspiration mode. To calibrate the injection needle make dot marks on the needle every 0.5 mm, which correspond to a volume of 50 nl.
4. For cytoplasmic injection, insert the tip of the needle into an oocyte in the vegetal hemisphere, very close to the animal hemisphere, at an approximately 45-degree angle. Turn the microinjector setting to microinject, and microinject each oocyte with 50 nl of import substrate. The dot marks on the needle are used to monitor the amount of substrate that has been injected.
5. Transfer the injected oocytes to a small (35-mm diameter) Petri dish filled with MBS, and incubate at room temperature for the desired amount of time (time points are chosen so as to allow observation of the import substrate associated with NPCs; this occurs between 10 and 30 minutes for proteins and for viruses that are actively transported toward the NPC; this time also depends on the site of injection and the size of the protein/virus).
6. When the incubation is complete, transfer oocytes to a 4-ml glass vial containing 2% glutaraldehyde in MBS, and fix overnight at 4 °C.

### Part 3: Dissection of *Xenopus* oocytes

1. The next day, wash the oocytes 3 times with MBS.
2. Transfer oocytes to a small Petri dish filled with low salt buffer (LSB: 1 mM KCl, 0.5 mM MgCl_2_, 10 mM HEPES, pH 7.5). Using a dissecting microscope and dissecting tweezers, remove the vegetal pole from each oocyte. It is useful to stabilize the oocyte being dissected with one pair of tweezers, while the other pair is used like scissors to remove the vegetal pole. At this point it is possible to evaluate the success of the microinjection by the presence of a blue tinge in the cytosol. The protocol is continued only for the oocytes that were microinjected successfully. In addition, if the samples are to be prepared for EM, this dissection step makes it much easier to find the nucleus when trimming and sectioning the samples.
3. Fix the dissected oocytes again with 2% glutaraldehyde in LSB for one hour at room temperature.
4. After fixation, wash the dissected oocytes three times with LSB.

### Part 4: Preparation of Injected Oocytes for Embedding and Thin-Sectioning EM

1. Transfer the dissected oocytes to a depression slide. Aspirate as much of the liquid as possible, and then quickly (so that they do not dry out) cover the dissected oocytes with 2% low-melting agarose. While the agarose is still soft, use a pipette tip to separate the oocytes from each other, and to ensure that each oocyte is face up or down (not sideways).
2. Allow the agarose to solidify for about 10 minutes. Once the agarose solidifies, use a razor blade to cut the agarose into small pieces, each containing one dissected oocyte.
3. Place the agarose-embedded oocytes in a 4-ml glass vial, and post-fix them with 1% OsO_4_ in LSB for one hour at room temperature.
4. Wash the sample three times in LSB. If necessary, store the sample at 4 °C overnight and continue the protocol the next day.
5. Sequentially dehydrate the samples in an ascending series of alcohol (50, 70, and 90% ethanol for 20 min each). This is followed by two changes in 100% ethanol for 15 min each. Finally dehydrate samples with 100% acetone for 15 min.
6. Infiltrate samples with a 1:1 mixture of Epon (Fluka) and acetone for one hour, followed by infiltration with a 2:1 mixture of Epon and acetone for two hours, and finally in pure Epon for at least six hours.
7. Place samples in flat embedding molds filled with fresh pure Epon. The oocytes are oriented in such a way that the side of the nucleus closer to the injection site – in this case the side of the oocytes that has been dissected – will be sectioned first. This step optimizes the chance of visualizing import of the chosen substrate.
8. Finally, polymerize the Epon for two days at 60 °C.
9. In order to visualize the samples, the blocks are trimmed and then sectioned using an ultramicrotome. Sections are transferred onto EM grids, stained using standard procedure, and visualized with a transmission electron microscope. *We will not show this part of the protocol as it is standard EM procedure*.

### Part 5: Representative Results:

If the protocol has been successful, then the nuclear envelope (NE) and NPCs should be clearly visible in EM micrographs. Depending on the substrate injected and the amount of time between injection and fixation, the substrate should be visible at the cytoplasmic face of the NPC, in the NPC, or at the nuclear face of the NPC.

Figure 1 shows a NE cross-section with adjacent cytoplasm (c) and nucleus (n) from a *Xenopus* oocyte that has been injected with capsids of the baculovirus AcMNPV, incubated at 4°C for four hours, and processed for embedding and thin section EM as described. Arrowheads point to NPCs. A capsid docking at the cytoplasmic face of a NPC is indicated by a white arrow.

In contrast, Figure 2 shows a NE cross-section with adjacent cytoplasm (c) and nucleus (n) from a *Xenopus* oocyte that has been injected with the parvovirus Minute Virus of Mice (MVM), incubated at room temperature for four hours, and processed for embedding and thin section EM as described. Using this technique, we have found that MVM induces disruptions of the NE (Cohen and Panté, 2005). Brackets indicate breaks in the NE. Arrowheads point to NPCs. Putative MVM capsids associated with the NE are indicated by white arrows.


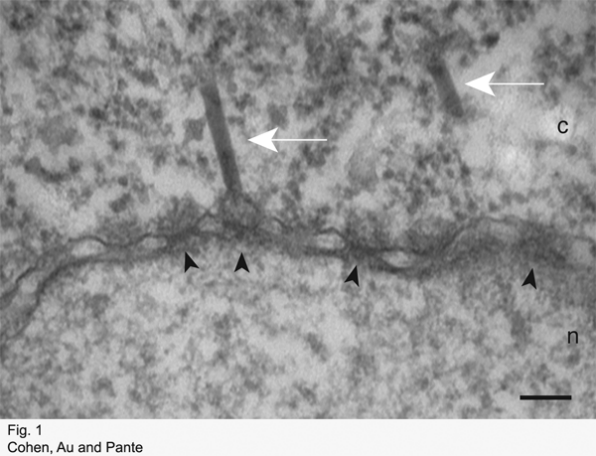


**Figure 1:** Electron micrograph of a NE cross-section with adjacent cytoplasm (c) and nucleus (n) from a *Xenopus* oocyte that has been injected with capsids of the baculovirus *Ac*MNPV, incubated at 4°C for four hours, and processed for embedding and thin section EM as described. Arrowheads point to NPCs. Capsids are indicated by white arrows. Scale bar: 100 nm.


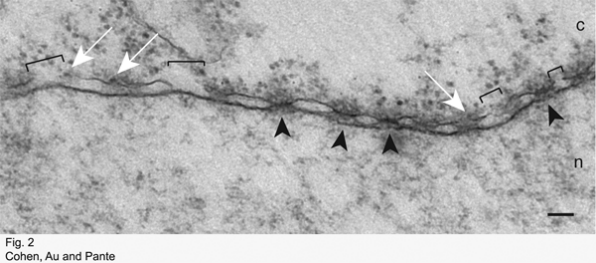


**Figure 2:** Electron micrograph of a NE cross-section with adjacent cytoplasm (c) and nucleus (n) from a *Xenopus* oocyte that has been injected with the parvovirus Minute Virus of Mice (MVM), incubated at room temperature for four hours, and processed for embedding and thin section EM as described. Arrowheads point to NPCs. Brackets indicate breaks in the NE. Putative MVM capsids associated with the NE are indicated by white arrows. Scale bar: 100 nm.

## Discussion

Microinjection of *Xenopus* oocytes combined with thin-sectioning EM is a highly effective tool for studying nucleocytoplasmic transport. This system has been used to map distinct steps of import through the NPC, for example interaction of a nuclear import substrate with structural components of the NPC such as the cytoplasmic filaments and nuclear basket (reviewed by Panté, 2006). It has also been used to study the nuclear import of nuclear-replicating viruses (Panté and Kann, 2002; Rabe *et al.*, 2003; Cohen and Panté, 2005).

One variation of the cytoplasmic microinjection technique shown here is nuclear microinjection of a nuclear export substrate (Panté *et al.*, 1997). To perform nuclear injection, oocytes are placed in a microwell plate, oriented so that the animal poles face up, and incubated at 4 °C overnight. The next day, the nuclei should be visible as a slightly raised area in the animal pole. The nuclei are microinjected with approximately 25 nl of export substrate at a steep (larger than 45-degree) angle and processed for embedding and thin-sectioning EM as described here.

It is also important to note that when dealing with a transport substrate that is not directly visible under the electron microscope, it is often necessary to label the substrate with an electron-opaque particle such as colloidal gold (reviewed by Panté, 2006).
